# Using postal change-of-address data to predict second waves in infections near pandemic epicentres

**DOI:** 10.1017/S0950268822000486

**Published:** 2022-03-24

**Authors:** Adam Schulman, Gyan Bhanot

**Affiliations:** 1 Rutgers Robert Wood Johnson Medical School, New Brunswick, NJ 08901, USA; 2 Department of Physics and Astronomy, Rutgers University, Piscataway, NJ 08854, USA; 3 Department of Molecular Biology and Biochemistry, Rutgers University, Piscataway, NJ 08854, USA; 4 Rutgers Cancer Institute of New Jersey, New Brunswick, NJ 08903, USA

**Keywords:** Covid-19, household migration and second wave in NY tristate area

## Abstract

We propose that postal Change-of-Address (CoA) data can be used to monitor/predict likely second wave caseloads in viral infections around urban epicentres. To illustrate the idea, we focus on the tri-state area consisting of New York City (NYC) and surrounding counties in New York, New Jersey and Connecticut States. NYC was an early epicentre of the coronavirus disease 2019 (Covid-19) pandemic, with a first peak in daily cases in early April 2020, followed by the second peak in May/June 2020. Using CoA data from the US Postal Service (USPS), we show that, despite a quarantine mandate, there was a large net movement of households from NYC to surrounding counties in the period April–June 2020. This net outward migration of households was strongly correlated with both the timing and the number of cases in the second peaks in Covid-19 cases in the surrounding counties. The timing of the second peak was also correlated with the distance of the county from NYC, suggesting that this was a directed flow and not random diffusion. Our analysis shows that CoA data is a useful method in tracking the spread of an infectious pandemic agent from urban epicentres.

## Introduction

Coronaviruses are large, enveloped, single-stranded RNA viruses. Although widespread in animals, they usually cause mild respiratory illnesses in humans [1–4]. In 2003, a new coronavirus emerged, named severe acute respiratory syndrome –corona virus (SARS-CoV), which caused life-threatening respiratory disease in humans with a fatality rate of ~10% [5, 6]. Since it impacted only a few countries and was quickly brought under control, interest in the development of treatment options and vaccines quickly waned. However, in late 2019, a second coronavirus, named severe acute respiratory syndrome coronavirus 2 (SARS-CoV-2), appeared in Wuhan, China. This virus has since caused a worldwide pandemic which is still ongoing [7–13]. SARS-CoV-2 is the seventh known coronavirus to cause pathology in humans [1]. The associated respiratory illness, called coronavirus disease 2019 (Covid-19), ranges in severity from a symptomless infection [7], to common-cold like symptoms, to viral pneumonia, organ failure, neurological complications and death [8–10]. While mortality rates from SARS-CoV-2 infections are significantly lower than from SARS-CoV [8–10], it has more favourable transmission characteristics, a higher reproduction number [12, 13] and a long incubation period when the patient may be asymptomatic but infective [14].

A large amount of consistent worldwide public data at varying granularity is available for viral sequences (https://www.ncbi.nlm.nih.gov/sars-cov-2/), number of tests performed, confirmed cases and deaths, including location, comorbidity and complications (https://Ourworldindata.org/coronavirus; https://github.com/CSSEGISandData/COVID-19/tree/master/csse_covid_19_data/csse_covid_19_time_series), and more recently, vaccine data (https://ourworldindata.org/covid-vaccinations?country=USA). These data are useful in modelling pandemic characteristics and evolving viral strains, which are important in guiding policy by predicting the potential impact of various interventions [15, 16]. For example, it is well known that the count of confirmed cases seriously underestimates the actual number of infections [17, 18], because not everyone who is infected is symptomatic or tested, and hence not everyone who dies from the disease is necessarily identified. The number of reported deaths are likely underestimated because of co-mortalities, (Covid-19 increases susceptibility to other diseases), lack of data from rural communities etc. Since this virus is also transmitted by asymptomatic individuals, who are a significant fraction of the infected population [19], it is difficult to make accurate estimates of transmission probabilities [20].

The World Health Organization identified contact tracing of infected individuals, followed by quarantining their contacts, as a key method to limit the spread of Covid-19 (https://www.who.int/publications/i/item/contact-tracing-in-the-context-of-covid-19). In places such as Singapore, South Korea, Thailand and China, where it was effectively implemented, it had an impressive impact [21, 22]. Some countries (e.g. Singapore and Australia) employed smartphone apps and QR codes to create ‘social monitoring’ programs to track movements of individuals to determine quarantine compliance, while others (e.g. countries in the European Union) have employed deidentified, encrypted Bluetooth tracking systems to identify epicentres of viral outbreaks [23–26]. Drones have also been used to monitor and track Covid-19 spread, deliver supplies and sanitise areas [27, 28]. However, for a variety of reasons, contact tracing was not implemented effectively everywhere [29]. In the United States, although strongly advocated by the CDC, the use of contract tracing was limited (https://www.cdc.gov/coronavirus/2019-ncov/php/contact-tracing/contact-tracing-resources.html; https://www.cdc.gov/mmwr/volumes/70/wr/mm7003a3.htm; https://www.pewresearch.org/internet/2020/10/30/the-challenges-of-contact-tracing-as-u-s-battles-covid-19/). These uncertainties suggest that methods to predict potential increases in rates of infection during an ongoing pandemic would be valuable.

The first Covid-19 case was confirmed in the United States on 21 January 2020, and New York City and the surrounding tri-state areas were early epicentres of the pandemic (https://www.investopedia.com/historical-timeline-of-covid-19-in-new-york-city-5071986), with the first confirmed cases identified in New York (NY) on 1 March, in New Jersey (NJ) on 4 March and in Connecticut (CT) on 8 March. The tri-states responded by declaring a state of emergency and issuing a joint mandate to stop the spread of the virus, including curfews, banning of large crowds and limiting access to bars, restaurants, gyms, movie theatres and casinos. As the virus continued to spread, a full-scale quarantine ‘stay at home order’ was issued and schools and non-essential businesses were closed. All three states stayed in these lockdown conditions until a phased reopening began with Phase 1 (NY on 8 June, NJ on 10 June, CT on 20 May) followed by several additional phases extending through June and July 2020 (https://www.njspotlight.com/2020/09/coronavirus-in-nj-the-first-six-months/; https://www.fox61.com/article/news/health/coronavirus/covid-19-timeline-see-how-fast-things-have-changed-in-connecticut/520-42ca1d55-c62a-4174-a2fe-40afde2fd495; https://abcnews.go.com/US/News/timeline-100-days-york-gov-andrew-cuomos-covid/story?id=71292880).

Despite these mandates, as we will show, from March–June 2020, there was a significant movement of households from New York City (NYC), which consist of the boroughs of The Bronx, New York (Manhattan), Kings (Brooklyn), Queens and Richmond (Staten Island) to the surrounding counties in the tri-state area. This movement of households can be tracked using ‘Change of Addresses’ or ‘CoA’ data, which is available on request obtained from the United States Postal Service (USPS) under the 1967 ‘Freedom of Information Act’.

In this paper, we show that CoA data is a useful data modality to track the spread of viral agents from early epicentres. We found that Excess CoA and county population density were strongly predictive of both the timing and total caseloads in the second peaks in Covid-19 cases in the tri-state counties surrounding New York City. Higher migration into the county (Excess CoA) brought in diseased individuals to spread the virus, and a higher county density made it more likely that they would meet people to infect. We also found that the time between the first and second peak in daily cases was positively correlated with the distance of the county seat from New York Penn Station, suggesting that the migration of the disease was a directed process (sudden movement of people from one location to another) and not diffusion.

## Methods

### Data sources and collection

- Postal data on address changes was obtained from the United States Postal Service (USPS) under the Freedom of Information Act (FOIA) using the portal at pfiapal.usps.com. We requested information on ‘change of address for people moving between New York City and New Jersey/New York/Connecticut States between 1 January 2019, until the present day’. The request was received on 4 January 2021, and labelled ‘No. 2021-FPRO-00700’. It was routed to the National Customer Support Centre on 5 January 2021, and fulfilled on 8 January 2021. The data provided by the USPS included monthly Change of Addresses (CoA) counts for households moving between NYC boroughs and each county in NJ, CT and NY State in both directions (Supplementary Tables 1a and 1b (https://drive.google.com/drive/folders/1hUBQVgeqzFs5IQ76qEGFFT1YvcXBwpiT?usp=sharing)). Monthly CoA under 10 households in a county was not provided by the USPS to avoid potential identification of specific households, which would violate USPS privacy policies.
- Data on Covid-19 Cases was obtained from: https://github.com/CSSEGISandData/COVID-19/tree/master/csse_covid_19_data/csse_covid_19_time_series and restricted to the three tri-state counties and the NYC boroughs of the Bronx, Kings (Brooklyn), New York City (Manhattan), Queens and Richmond (Staten Island).
- Population data for 2019 for each tri-state county was obtained from:
  https://www.census.gov/data/tables/time-series/demo/popest/2010s-counties-total.html.
- Data on county size was obtained from the following:
  NJ: https://www.indexmundi.com/facts/united-states/quick-facts/new-jersey/land-area#table
  CT: https://www.indexmundi.com/facts/united-states/quick-facts/connecticut/land-area#table
  NY: https://www.indexmundi.com/facts/united-states/quick-facts/new-york/land-area#table

### Model for the initial peak in Covid-19 cases in each county

Caseloads in the first peak of Covid-19 cases in each county were estimated using a SIR model, which was previously proposed and applied to study the early phase of the Covid-19 daily cases and deaths in eight European countries and the United Kingdom [30]. We present below a brief outline of the model. Further details are in [30].

The basic variables of the model are:1
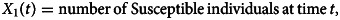
2
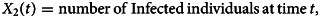
3
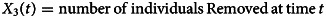
4
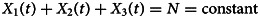
and the basic equations we solve are:5
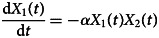
6
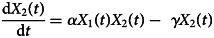
with boundary conditions defined at some time *t* = *t*_0_ in the early stages of the pandemic:7
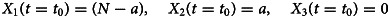


The pandemic parameter *R* is given by:8
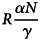


Since the four parameters *α*,  *γ N* and *R* are related by Eq. 8, only three need to be estimated from the data. These were determined using the following procedure applied to each county (details in Supplementary Appendix A):

- The data for *X*_2_(*t*) was smoothed by averaging the daily cases over 7 days.
- Using the smoothed data, *γ*(*R*−1) was estimated from the exponent of *X*_2_(*t*) for small *t*, using the result small *t*: *X*_2_(*t*) ~ e^*γ*(*R*−1)*t*^ (Eq. A17). The time point where the fitting procedure was started was chosen so that ‘a’ in Eq. 7 had a minimum value of 5–10. Starting the fits a few days after this time point had no effect on the fitted value of *γ*(*R*−1). This procedure eliminates one variable (*γ*).
- Using the Matlab solver ode45, the measured maximum value of the first *X*_2_ peak for each county, and the exact result: maximum value of *X*_2_ ≡ *P* = *N* − (*N*/*R*)[ 1+ log (*R*)] (Eq. A11), R was varied to find *α* to fit the data up to and past the first peak.

Note that in this procedure, the only least squares ‘fit’ is in the estimate of *γ*(*R*−1). Perturbing the data for *X*_2_ in the region of the ascending limb showed that *γ*(*R*−1) was very accurately measured, with a very small confidence interval. Thus, given the data for *X*_2_, the error from the fitting procedure described above had a negligible effect on N_CS_.

An additional source of error in N_CS_ is from variations in the daily counts, i.e. from stochastic fluctuations in the data for *X*_2_. This error was estimated using the following procedure:

- For each non-zero value of *X*_2_(*t*) and for each time point *t*, 50 Gaussian values were generated, with mean and variance *X*_2_(*t*). This generated 50 perturbed datasets for each county.
- For each of these perturbed datasets, using the fitted values of the parameters from the original data, N_CS_ values were recomputed to find an average value of N_CS_ and its variation (standard deviation) ΔN_CS_. These values are reported in Table 1 and were used in the subsequent analysis reported below.

**Table 1.** Summary of results for each county

State/County	P	D	N_cs_	ΔN_cs_	E_coA_
NJ/ATLANTIC	474	127	2132	45	370
NJ/BERGEN	4001	15	6166	250	3810
NJ/BURLINGTON	558	78	3272	109	332
NJ/CAMDEN	2289	100	4719	154	347
NJ/CAPEMAY	366	148	467	35	314
NJ/CUMBERLAND	309	131	2745	50	11
NJ/ESSEX	6331	14	8404	319	2381
NJ/GLOUCESIER	906	100	1643	70	0
NJ/HUDSON	14 557	5	9146	290	2803
NJ/HUNTERDON	291	60	592	32	348
NJ/MERCER	1636	68	4709	141	734
NJ/MIDDLESEX	2671	38	8417	205	1430
NJ/MONMOUTH	1320	49	3873	78	2983
NJ/MORRIS	1069	35	2590	137	1059
NJ/OCEAN	966	76	4914	108	1289
NJ/PASSAIC	2719	20	9244	206	626
NJ/SALEM	188	126	552	26	#N/A
NJ/SOMERSET	1090	47	1928	97	675
NJ/SUSSEX	271	60	239	30	359
NJ/UNION	5409	17	7914	177	1279
NJ/WARREN	296	72	632	70	67
CT/F AIRFIELD	1509.6	61	9190	216	5545
CT/HARTFORD	1213.1	115	4463	153	1031
CT/LITCHFIELD	195.9	98	0	0	2253
CT/MIDDLESEX	439.8	103	705	73	399
CT/NEW HAVEN	1414.0	80	3788	109	1409
CT/NEW LONDON	398.9	126	611	53	585
CT/TOLLAND	367.4	128	182	27	77
CT/WINDHAM	227.7	140	161	20	#N/A
NY/NASSAU	4765.8	22	6120	304	7379
NY/WESTCHESTER	2247.4	30	19 526	183	6118
NY/ROCKLAND	1877.2	34	1922	69	1047
NY/SUFFOLK	1619.0	75	13 857	261	11 771
NY/ORANGE	474.2	62	3134	80	1310
NY/PUTNAM	426.9	59.1	0	0	989
NY/DUTCHESS	369.8	81.4	1919	121	2243
NY/ULSTER	157.9	96.3	0	0	3030
NY/SULLIVAN	77.9	92.7	382	56	1322

P, County Population Density (# persons/square mile), D, Distance of County Seat from New York Penn Station (miles), N_CS_, Number of cases in the second peak, ΔN_CS_, error estimate for N_CS_, E_CoA_, Excess Change of Addresses from NYC into the county.

## Results

### Excess migration from NYC to tri-state area, March–June 2020 *vs.* 2019

**Fig. 1.**
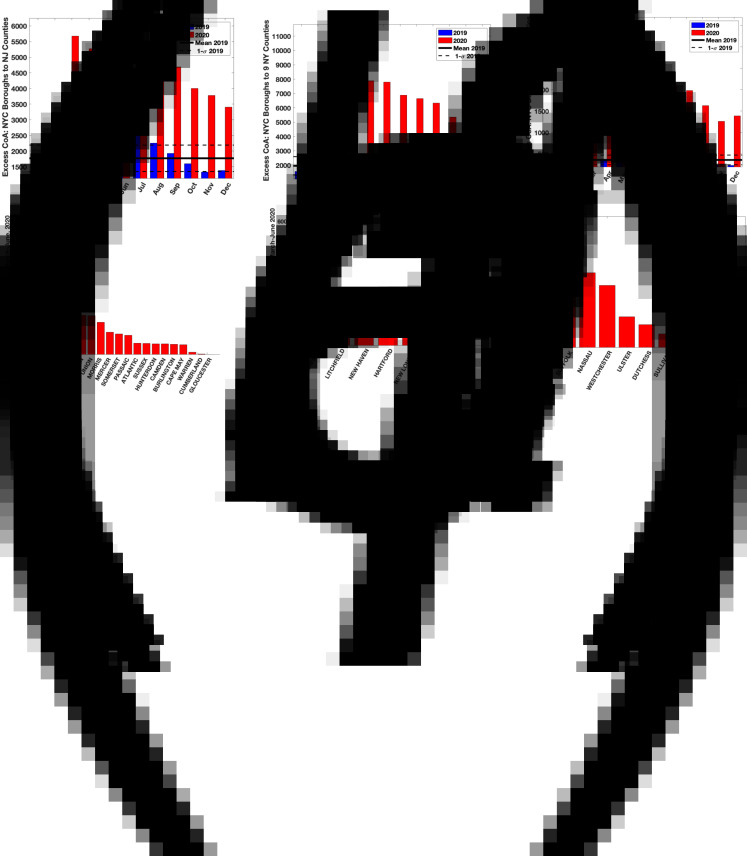
(a–c) LtoR top: Monthly Excess Change of Address (CoA) counts from New York City boroughs to all counties in New Jersey and Connecticut and 9 tri-state counties (Suffolk, Nassau, Westchester, Ulster, Dutchess, Orange, Sullivan, Rockland and Putnam) in New York State in 2019 (blue bars) and 2020 (red bars). The solid horizontal and dashed lines represent the average and one-sigma limits for Excess CoA for the twelve months in 2019. (d–f) LtoR bottom: Cumulative Excess Change of Address (CoA) counts from New York City boroughs to each county in New Jersey and Connecticut and 9 tri-state counties (Suffolk, Nassau, Westchester, Ulster, Dutchess, Orange, Sullivan, Rockland and Putnam) in New York State for the months of March–June 2020.

Figures 1a–c shows the excess number of household moves [Changes of Addresses (CoA)] for each month in 2019 and 2020 from NYC to NJ, CT and nine NY counties in the tri-state area closest to NYC (map in Supplementary Fig. SF1). Figures 1d–f shows the cumulative excess CoA from March–June 2020 from NYC into each county in NJ, CT and nine tri-state counties in NY, namely Suffolk, Nassau, Westchester, Ulster, Dutchess, Orange, Sullivan, Rockland and Putnam. Note that Windham County in CT is omitted from Figure 1e because there were fewer than 10 household moves in either direction for this county in 2020. Additional figures, available here (https://drive.google.com/drive/folders/1P7McgYj-wjCYlrjGQXbrLVYC_vqwWRqt?usp=sharing) show CoA from each NYC borough into each county in 2019 and 2020 by month, showing clear increases in household moves from March 2020 onwards compared to 2019. Overall, these results demonstrate that from March 2020 onwards, compared to the same periods in 2019, there was a significant net migration of households out of NYC boroughs into surrounding counties in NJ, CT and NY.

### Cases in the first and second peaks in daily Covid-19 cases in each county

**Fig. 2.**
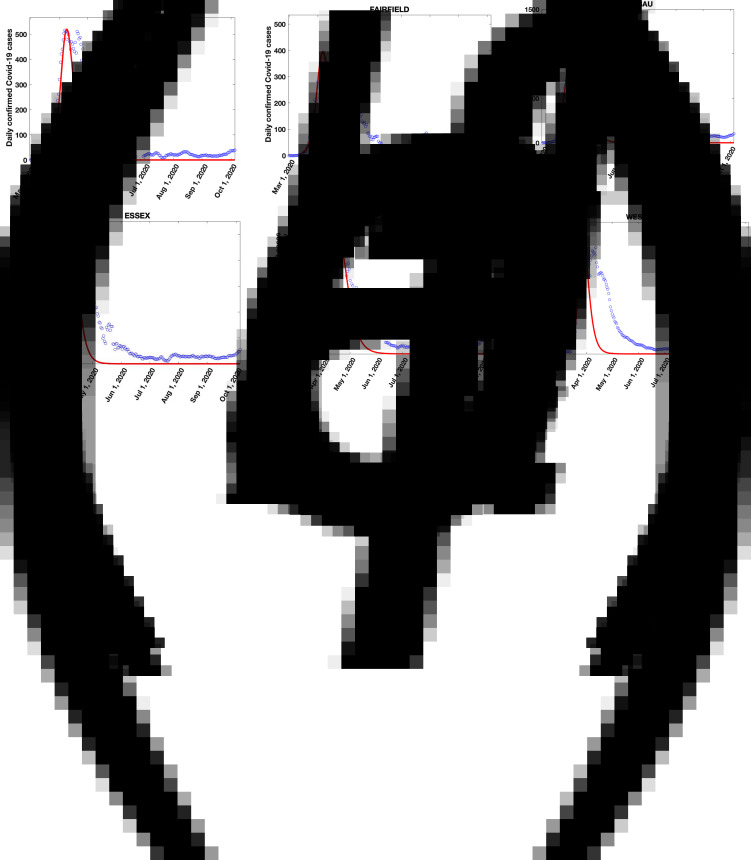
(a–f) LtoR top, LtoR bottom: Seven-day averaged daily Covid-19 cases (blue circles) in the two counties with the highest population densities in each of the tri-states: Hudson and Essex in NJ, Fairfield and New Haven in CT and Nassau and Westchester in NY. The solid red curves are fits to the data for the first peak from a SIR model (Methods and [30]).

Figures 2a–f shows the seven-day averaged plots of daily Covid-19 cases in the two counties with the highest population densities in each of the tri-states: Hudson and Essex in NJ, Fairfield and New Haven in CT and Nassau and Westchester in NY. Similar plots for the two counties with the next highest population density in each state are shown in Supplementary Figs 2a–f: Union and Bergen in NJ, Hartford and Middlesex (CT) in CT and Rockland and Suffolk in NY. Results for all counties in NJ, CT and the NY counties in the tri-state area are available here (https://drive.google.com/drive/folders/1SgjBDGIej1vIz1joPJEsTJXnWdSMXvqH?usp=sharing).

**Fig. 3.**
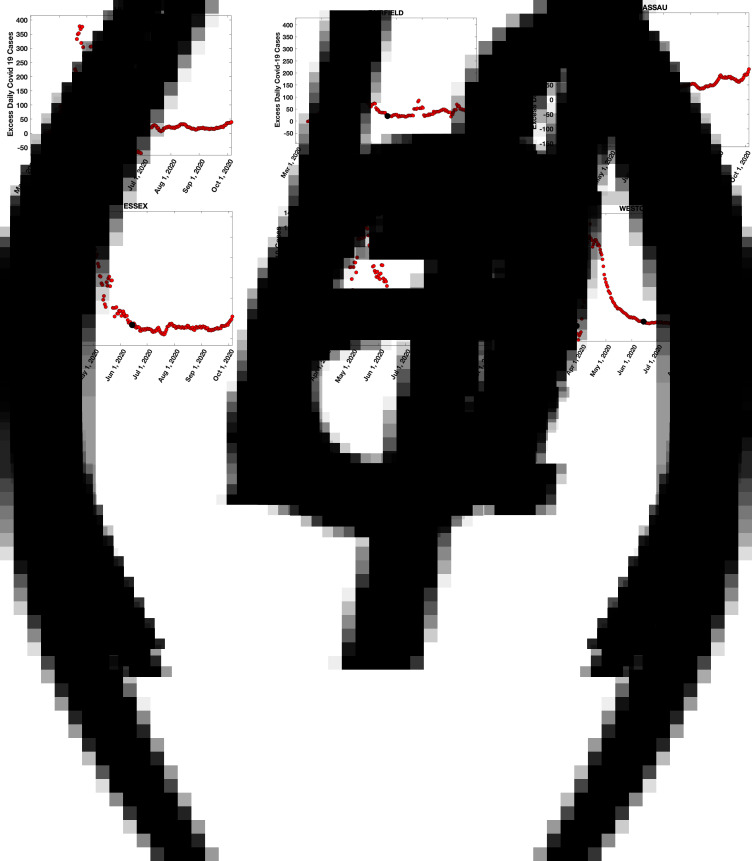
(a–f) LtoR top, LtoR bottom: Daily cases in the second peak, obtained from data for Figures 2a–f by subtracting the SIR model predictions (solid red line) from the seven-day averaged data (blue circles). The number of cases N_CS_ in the second peak was estimated by adding the daily case data between the black dots, which approximate the timepoints of the start and end of the second peak. The first time point was chosen as the day the second peak daily case load was approximately zero and the second time point when it tapered off to a constant value.

These results show that there was an initial peak in daily cases in all the tri-state counties in early April 2020. After the first peak, in every county, data for daily cases had either a shoulder or a clear second peak. To identify the number of cases in the shoulder or second peak, we subtracted the expected daily cases in the first peak using the SIR model described in Methods ([30] has additional details). The model fits are shown in Figure 2 and Supplementary Fig. 2 as solid red lines. Subtracting the model fits from the daily case data of Figures 2a–f and Supplementary Figs 2a–f results in the plots of Figures 3a–f and Supplementary Figs 3a–f, which show the daily cases in the second peak (plots for all counties in NJ, CT and the NY counties in the tri-state area are here (https://drive.google.com/drive/folders/1wPlLJ6msW59b0DgoeixXwAtpBlYnYZpV?usp=sharing). From these results, we estimated the number of cases in the second peak for each county by summing the second peak data for daily cases between the time points shown as black dots, which were chosen on the left as the point when the daily case count in the second peak was approximately zero and on the right when the daily case count in the second peak became approximately constant (and small compared to the peak).

### Cases in the second peak correlate with county population density and excess CoA influx

**Fig. 4.**
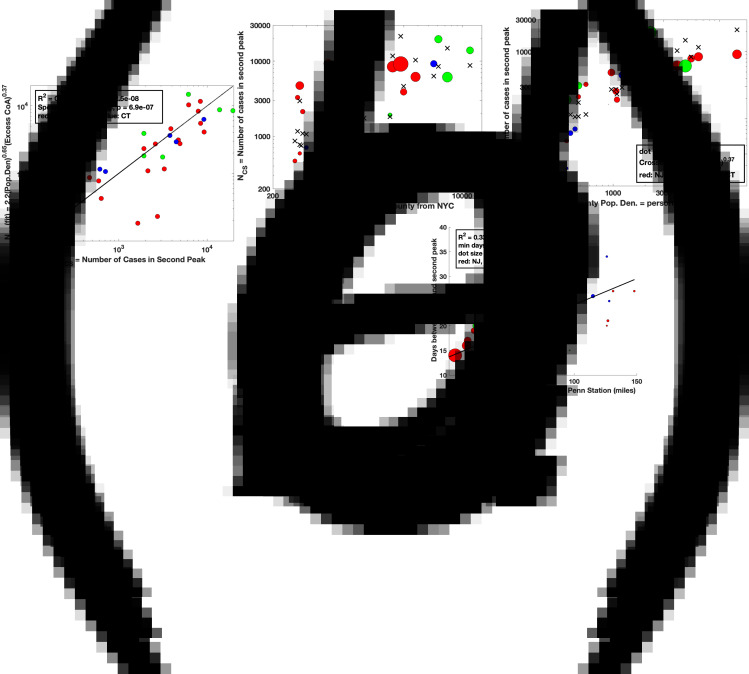
(a) Bivariate linear regression fit to the model: 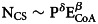 where N_CS_ is the number of cases in the second peak in a given county, P is the population density in the county and E_CoA_ is the excess households that moved into the county from NYC from March–June 2020. (b) Projection of the fit of (a) to the *P* axis. (c) Projection of the fit of (a) to the E_CoA_ axis. (d) Number of days from the first peak in daily cases to the second peak for each county versus the distance by road of the county seat from NY Penn Station (miles). Note that the minimum number of days between peaks (intercept on *y* axis) is 13.7 days, which is possibly related to the quarantine duration mandate for infected people in the first peak and to the latency between infection and symptoms.

If migration of households contributed to the second peak in Covid-19 cases, the number of cases in the second peak should be correlated with the excess CoA and the county population density. We found that the data best fits to the form: 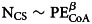 where N_CS_ = the number of cases in the second peak, P is the county population density, and E_CoA_ is the excess CoA into the county from NYC. Figures 4a–c shows the results of the fit with *δ* = 0.65 and *β* = 0.37, *R*^2^ = 0.74, *F* = 37.0, *P*-value = 2.5 × 10^−8^, Spearman Rank Correlation = 0.88, *P*-value = 6.9 × 10^−7^.

We also find (Fig. 4d) that the number of days from the first to the second peak is linearly correlated with the distance of the county seat from NY Penn Station (Fig. 4b, *R*^2^ = 0.33, *F* = 16.9, *P*-value = 2.3 × 10^−4^, Pearson Correlation = 0.61, *P*-value = 7.0 × 10^−5^). The linear relationship between time and distance suggests a directed migration and not a random diffusion of people. This observation supports our hypothesis that the excess CoA from NYC to the tri-state area likely contributed to the second peak. The intercept of 13.7 days on the *y*-axis in Figure 4d is likely related partly to the 14-day quarantine imposed in NYC for symptomatic individuals, which prevented infected individuals from moving out of NYC early in the pandemic, and partly from the latency of infection (*L* = 1/*γ*) the period when individuals are infected and can infect others but are not yet symptomatic themselves. Table 1 contains a summary of all our results.

## Discussion

We show evidence in support of the hypothesis that the migration of people from NYC to counties in the surrounding tri-state area from March–June 2020 contributed to the Covid-19 cases in the second peak of infections in these counties. Excess migration of people from NYC to each county was computed using Change of Address (CoA) data obtained from the United States Postal Service. To model the first peak in cases, we used a simple SIR model (Methods, [30]) which, when subtracted from the daily case data, allowed us to identify the daily Covid-19 cases in the second peak. We found that the number of Covid-19 cases in the second peak correlated with both the excess CoA from NYC into the counties and the county population density (Figs 4a–c). Furthermore, the time between the first and second peaks in each county was proportional to the distance of the county seat from NY Penn Station (Fig. 4d), suggesting that the spread of disease was due to directed migration (CoA related) and not diffusion.

It is interesting to speculate on the types of households that moved out of the NYC boroughs into the suburbs. One possibility is that it was just households that normally move out of the city each year to stay in their summer homes and then return to NYC at the end of the summer. However, this is unlikely to be the explanation because the total number of people moving out was significantly higher in 2020 compared to 2019, the moves happened before the end of the school year and did not end with the end of summer (Fig. 1). It is more likely that, because of the panic induced by the pandemic, many households sold their homes or broke their rental leases in NYC, and either bought or rented homes in the suburbs. This migration seems to have completely escaped the attention of the media.

We now list some caveats and limitations of our study, some of which can be mitigated by additional data and future research:

The USPS data did not provide household moves less than 10 per month. However, the average excess CoA from the 15 tri-state counties with the highest population density was 298 changes per month from March–June 2020. Consequently, although data for some smaller rural counties may have been missed in our analysis, we do not expect this to affect our results, because our correlation results are derived from more populated counties, with hundreds to thousands of excess household moves (Table 1 and Fig. 4b). A more serious limitation was the fact that the data only reported movements of ‘households’ and did not specify the average number of people per household. If CoA data is used in tracking pandemics in the future, it may be possible to correct this limitation by using the average household size in a county or by using cell phone data, possibly with voice tagging.

Our analysis did not account for the fact that many people commute into NYC each day from the tri-state counties (https://www.census.gov/topics/employment/commuting/guidance/flows.html), especially from Westchester, Nassau, Suffolk Counties in NY, Bergen, Essex, Middlesex and Hudson Counties in NJ and Fairfield County in CT (see columns P and Q in Table 1). It is possible that when households relocated out of NYC into these counties, some individuals in these households continued to commute into the city daily because of their jobs. Such individuals would increase the risk of Covid-19 infections spreading into those counties. It is possible to include this correction into our model by studying commuter traffic data between NYC and the surrounding counties in the period March–June 2020. Unfortunately, this data is not yet available. Such an analysis would also have to correct for Work from Home (WfH) effects which allowed many workers to avoid their commute.

Another important limitation of our study is that we do not have data on infection rates among the people who moved. To our knowledge, accurate data detailing the movement of infected individuals does not exist, although confirmed hospital admissions of Covid-19 cases and tracking of their prior movements may provide such data. We note however, that the virus spread more pervasively in NYC residents compared to those in surrounding counties, because there was a higher number of daily cases during the first peak in NYC boroughs compared to tri-state counties (compare the height of the first peak in Supplementary Figs 3a–e to that in Figs 2a–f and Supplementary Figs 2a–f). This means that households moving out of NYC were more likely to contain infected individuals than households moving in the other direction. An interesting way to extend our study to simulate these effects would be to use the CoA data to seed infected individuals moving into various counties and use fits to the actual location and height of the second peaks to find the fractions of these infected individuals, using the number of cases in the first peak to estimate the initial values for these fractions.

Finally, the starting and end points of the second peak were defined in this study in a somewhat arbitrary manner (Figs 3a–f and Supplementary Figs 3a–f). However, changing the start and end points of the second peak by a few days in either direction did not change the number of cases in the second peak (N_CS_) significantly and hence do not affect our conclusions.

## Data Availability

All materials (data and codes) needed to replicate the findings of the article will be made available at a website once the paper is accepted.

## Supplementary material

For supplementary material accompanying this paper visit http://doi.org/10.1017/S0950268822000486. click here to view supplementary material

## Links mentioned in the paper

Link1: https://drive.google.com/drive/folders/1P7McgYj-wjCYlrjGQXbrLVYC_vqwWRqt?usp=sharing.

Link2: https://drive.google.com/drive/folders/1SgjBDGIej1vIz1joPJEsTJXnWdSMXvqH?usp=sharing.

Link3: https://drive.google.com/drive/folders/1wPlLJ6msW59b0DgoeixXwAtpBlYnYZpV?usp=sharing.
