# Supplementary figures and images for: Using postal change-of-address data to predict second waves in infections near pandemic epicentres
Source: Epidemiol Infect. 2022 Mar 24;150:e120. doi: 10.1017/S0950268822000486 (PMC9254154; doi:10.1017/S0950268822000486)

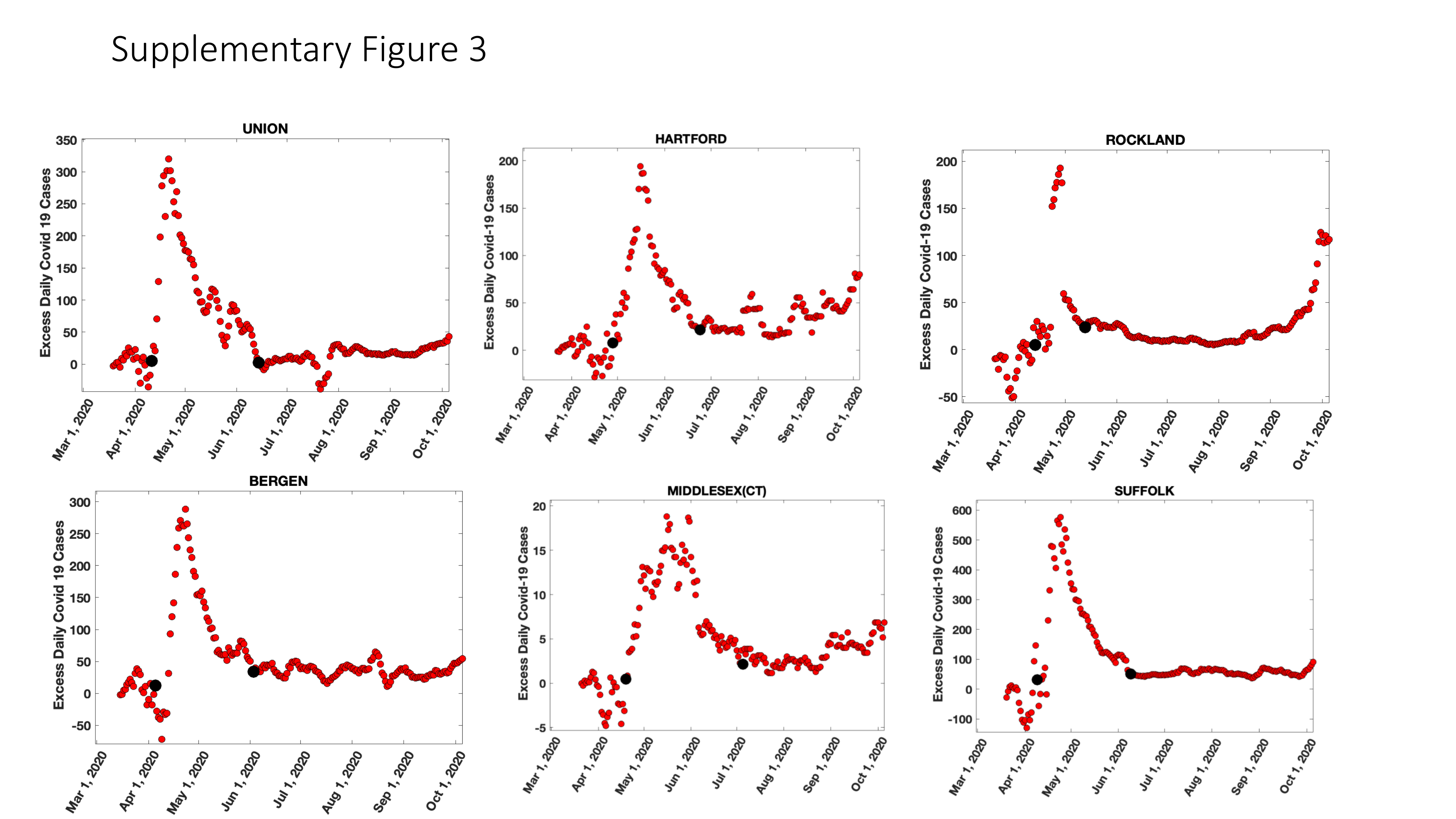

Supplement: Supplementary file 1 [file S0950268822000486sup001.zip › SF3.png]

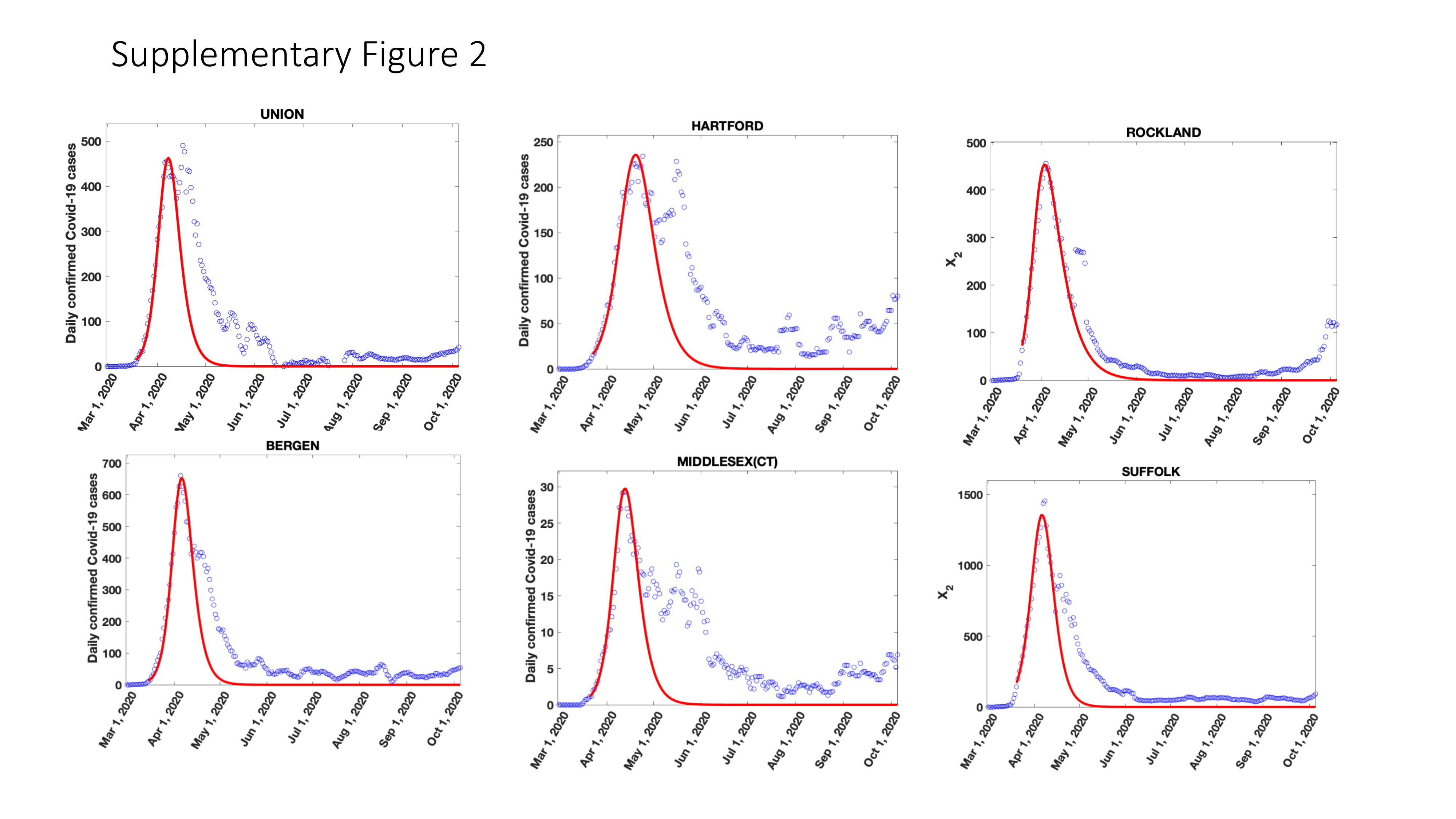

Supplement: Supplementary file 1 [file S0950268822000486sup001.zip › SF2.png]

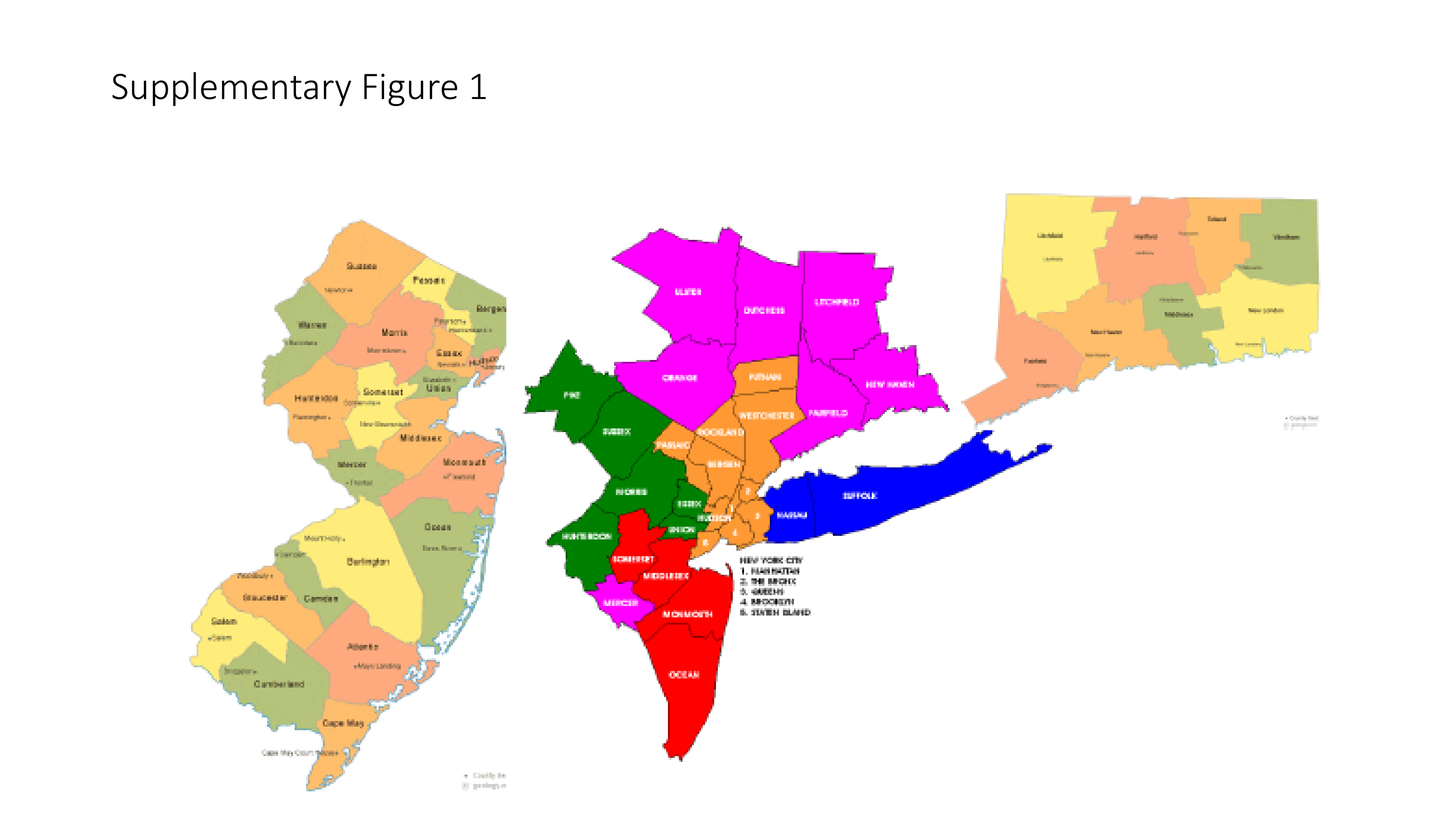

Supplement: Supplementary file 1 [file S0950268822000486sup001.zip › SF1.png]
